# Mitochondrial DNA 5178 C/A polymorphism modulates the effects of coffee consumption on elevated levels of serum liver enzymes in male Japanese health check-up examinees: an exploratory cross-sectional study

**DOI:** 10.1186/s40101-016-0098-2

**Published:** 2016-06-04

**Authors:** Akatsuki Kokaze, Masao Yoshida, Mamoru Ishikawa, Naomi Matsunaga, Kanae Karita, Hirotaka Ochiai, Takako Shirasawa, Hinako Nanri, Kiyomi Mitsui, Hiromi Hoshimo, Yutaka Takashima

**Affiliations:** 1 Department of Public Health, Showa University School of Medicine, 1-5-8 Hatanodai, Shinagawa-ku, Tokyo 142-8555 Japan; 2 Department of Public Health, Kyorin University School of Medicine, 6-20-2 Shinkawa, Mitaka-shi, Tokyo 181-8611 Japan; 3 3Mito Red Cross Hospital, 3-12-48 Sannomaru, Mito-shi, Ibaraki 310-0011 Japan

**Keywords:** Coffee consumption, Longevity, Mitochondrial DNA polymorphism, Personalized prevention, Serum liver enzymes

## Abstract

**Background:**

Longevity-associated mitochondrial DNA 5178 cytosine/adenine (Mt5178 C/A) polymorphism modulates the effects of coffee consumption on the risk of hypertension, dyslipidemia, and abnormal glucose tolerance. The objective of this study was to investigate whether Mt5178 C/A polymorphism modifies the effects of coffee consumption on abnormally elevated levels of serum liver enzymes in male Japanese health check-up examinees.

**Methods:**

A total of 421 male subjects (mean age ± SD, 54.1 ± 7.7 years) were selected from among individuals visiting the hospital for regular medical check-ups. After Mt5178 C/A genotyping, a cross-sectional study assessing the joint effects of Mt5178 C/A polymorphism and coffee consumption on elevated levels of serum aspartate aminotransferase (AST), serum alanine aminotransferase (ALT), and serum gamma-glutamyl transpeptidase (GGT) was then conducted.

**Results:**

For men with Mt5178C, after adjustment for age, body mass index, alcohol consumption, habitual smoking, green tea consumption, antihypertensive treatment, and antidiabetic treatment, elevated levels of serum AST, as defined as ≥30 U/L; those of serum ALT, as defined as ≥25 U/L; or those of serum GGT, as defined as ≥60 or >51 U/L, may depend on coffee consumption (*P* for trend = 0.013, *P* for trend <0.001, *P* for trend = 0.002, and *P* for trend <0.001, respectively). On the other hand, no significant joint effects of Mt5178A genotype and coffee consumption on elevated levels of serum liver enzymes were observed.

**Conclusions:**

The present results suggest that Mt5178 C/A polymorphism modifies the effects of coffee consumption on abnormally elevated levels of serum liver enzymes in male Japanese health check-up examinees.

## Background

Coffee is an increasingly popular beverage worldwide, and coffee intake has been reported to have beneficial effects on health [1, 2]. Recently, Saab et al. published a systematic review of studies on the effects of coffee consumption on liver cirrhosis, chronic hepatitis B and hepatitis C, non-alcoholic fatty liver disease (NAFLD), and hepatocellular carcinoma [3]. They recommend daily coffee consumption to patients with chronic liver disease. Their systematic review also summarized epidemiological surveys reporting the inverse association between coffee consumption and elevated levels of serum liver enzymes, namely serum aspartate aminotransferase (AST) [4, 5], serum alanine aminotransferase (ALT) [4–7], and serum gamma-glutamyl transpeptidase (GGT) [4, 8].

Mitochondrial DNA 5178 cytosine/adenine (Mt5178 C/A) polymorphism (rs28357984), also known as NADH dehydrogenase subunit-2 237 leucine/methionine (ND2-237 Leu/Met) polymorphism, is associated with longevity in the Japanese population [9]. The frequency of the Mt5178A genotype is significantly higher in Japanese centenarians than in the general population [9]. Japanese individuals with Mt5178A are more resistant to lifestyle-related adult onset diseases, such as hypertension [10], diabetes [11], myocardial infarction [12, 13], and cerebrovascular disorders [14], than those with Mt5178C. Mt5178 C/A polymorphism influences the effects of coffee consumption on the risk of hypertension [15], those of abnormal glucose tolerance [16], those of hyper-low-density lipoprotein (LDL) cholesterolemia [17], and those of anemia [18]. Moreover, this polymorphism modulates the effects of cigarette smoking on elevated levels of serum liver enzymes [19]. However, there has been no research on the gene-environment interactions between Mt5178 C/A polymorphism and coffee consumption on elevated levels of serum liver enzymes.

The objective of this study is to investigate whether there is a combined effect of longevity-associated Mt5178 C/A polymorphism and coffee consumption on abnormally elevated levels of serum liver enzymes in male Japanese health check-up examinees.

## Subjects and methods

### Subjects

Participants were recruited from among individuals visiting the Mito Red Cross Hospital for regular medical check-ups between August 1999 and August 2000. This study was conducted in accordance with the Declaration of Helsinki and was approved by the Ethics Committee of the Kyorin University School of Medicine. Written informed consent was obtained from 602 volunteers before participation. Because the number of women was insufficient for classification into groups based on Mt5178 C/A genotype and coffee consumption, female health check-up examinees were excluded. Male health check-up examinees with unclear clinical data were also excluded. Thus, 442 men were enrolled in the study. Twenty-one individuals with a history of chronic liver diseases, including hepatitis B and hepatitis C, were also excluded. Therefore, subjects comprised 421 Japanese men (age, 54.1 ± 7.7 years; mean ± SD).

### Clinical characteristics of subjects

Blood chemical and physical data, including serum AST, ALT, and GGT levels, in the present study were obtained from the results of regular medical check-ups [20]. Elevated levels of serum AST were defined as ≥30 U/L [21]. Elevated levels of serum ALT were defined as >30 U/L [22] or ≥25 U/L [23]. Elevated levels of serum GGT were defined as ≥60 U/L [24, 25] or >51 U/L [26, 27]. Body mass index (BMI) was defined as the ratio of subject weight (kg) to the square of subject height (m). Information on antihypertensive treatment or antidiabetic treatment was derived from health check-up records. A survey of coffee intake, alcohol consumption, and habitual smoking was performed by means of a questionnaire. Taking into account sample size in this study and others [7], coffee consumption was categorized based on the number of cups of coffee per day (<1 cup per day; 1–2 cups per day; and ≥3 cups per day). Green tea consumption was also categorized based on the number of cups of green tea per day (≤1 cup per day; 2–3 cups per day; and ≥4 cups per day). Alcohol consumption was classified based on drinking frequency (daily drinkers; occasional drinkers, including those who drink several times per week or per month; and non- or ex-drinkers). Smoking status was classified based on the number of cigarettes smoked per day (never- or ex-smokers; 1–20 cigarettes smoked per day; and >20 cigarettes smoked per day).

### Genotyping

DNA was extracted from white blood cells using the DNA Extractor WB kit (Wako Pure Chemical Industries, Osaka, Japan). Mt5178 C/A polymorphism was detected by PCR and digestion with *Alu*I restriction enzyme as described previously [10, 15–20]. Primers were forward 5′-CTTAGCATACTCCTCAATTACCC-3′ and reverse 5′-GTGAATTCTTCGATAATGGCCCA-3′. PCR was performed with 50 ng of genomic DNA in buffer containing 0.2 μmol/L of each primer, 1.25 mmol/L dNTPs, 1.5 mmol/L MgCl_2_, and 1 U of Taq DNA polymerase (GeneAmp, PerkinElmer, Branchburg, NJ, USA). After an initial denaturation at 94 °C for 5 min, PCR was conducted through 40 cycles in the following steps: denaturation at 94 °C for 30 s, annealing at 60 °C for 60 s, and polymerase extension at 72 °C for 90 s. After cycling, a final extension at 72 °C for 10 min was performed. PCR products were digested with *Alu*I restriction enzyme (Nippon Gene, Tokyo, Japan) at 37 °C overnight and were electrophoresed in 1.5 % agarose gels stained with ethidium bromide for visualization under ultraviolet light. The absence of an *Alu*I site was designated as Mt5178A, and the presence of this restriction site was designated as Mt5178C.

### Statistical analyses

Statistical analyses were performed using SAS statistical software version 9.4 for Windows. Multiple logistic regression analysis was used to calculate odds ratios (ORs) for abnormally elevated levels of serum liver enzymes (AST ≥30 U/L; ALT >30 or ≥25 U/L; GGT ≥60 or >51 U/L). For multiple logistic regression analysis, alcohol consumption (non- or ex-drinkers = 0; occasional drinkers, including those who drink several times per week or per month = 1; daily drinkers = 2), habitual smoking (non- or ex-smokers = 0; 1–20 cigarettes smoked per day = 1; >20 cigarettes smoked per day = 2), green tea consumption (≤1 cup per day = 1; 2–3 cups per day = 2; ≥4 cups per day = 2), antihypertensive treatment (subjects without antihypertensive treatment = 0; those under antihypertensive treatment = 1), and antidiabetic treatment (subjects without antidiabetic treatment = 0; those under antidiabetic treatment = 1) were numerically coded. Differences with *P* values of less than 0.05 were considered to be statistically significant.

## Results

Except for the AST/ALT ratio, no significant differences in clinical characteristics were observed between the Mt5178C and Mt5178A genotypes (Table 1). The AST/ALT ratio was significantly higher in subjects with Mt5178C than in those with Mt5178A (*P* = 0.004). Although the difference did not reach significance (*P* = 0.089), the frequency of subjects under antihypertensive treatment was higher in Mt5178C genotypic men than in Mt5178A genotypic men.

**Table 1:** Clinical characteristics of study subjects by Mt5178 C/A genotype

	Mt5178C	Mt5178A	*P* value
	*N* = 251	*N* = 170	
Age (years)^a^	54.5 (7.6)	53.6 (7.8)	0.245
BMI (kg/m^2^)^a^	23.1 (2.8)	23.5 (2.6)	0.103
SBP (mmHg)^b^	126.1 (16.0)	125.3 (13.9)	0.614
DBP (mmHg)^b^	73.9 (10.5)	73.5 (9.0)	0.628
AST (U/L)^c^	22 (18–27)	23 (19–27)	0.687
ALT (U/L)^c^	21 (15–32)	24 (18–35)	0.471
AST/ALT ratio^c^	1.037 (0.818–1.286)	0.918 (0.723–1.190)	0.004
GGT (U/L)^c^	42 (27–75)	47 (30–75)	0.427
TC (mg/dL)^a^	204.2 (31.5)	202.5 (31.9)	0.590
LDL-C (mg/dL)^a^	122.5 (30.4)	118.4 (31.7)	0.182
HDL-C (mg/dL)^b^	55.1 (13.6)	55.9 (15.7)	0.576
TG (mg/dL)^c^	112 (84–156)	113 (83–161)	0.378
Subjects with ≥140 mg/dL LDL-C (%)^d^	25.5	27.1	0.721
Subjects with ≥150 mg/dL TG (%)^d^	28.3	29.4	0.803
FPG (mg/dL)^c^	97 (92–105)	97 (91–103)	0.240
UA (mg/dL)^a^	5.93 (1.28)	5.98 (1.21)	0.686
Coffee consumption (<1 cup per day/1–2 cups per day/≥3 cups per day) (%)^d^	45.8/31.5/22.7	36.5/40.0/23.5	0.120
Green tea consumption (≤1 cup per day/2–3 cups per day/≥4 cups per day) (%)^d^	26.7/30.7/42.6	27.7/32.9/39.4	0.798
Alcohol consumption (non- or ex-/occasionally/daily drinkers) (%)^d^	17.9/34.3/47.8	12.9/40.0/47.1	0.285
Current smokers (%)^d^	58.6	58.2	0.946
Subjects under antihypertensive treatment (%)^d^	19.9	13.5	0.089
Subjects under antidiabetic treatment (%)^d^	7.2	7.7	0.855

Age, BMI, SBP, DBP, TC levels, LDL-C levels, HDL-C levels, and UA levels are given as means (S.D.). AST levels, ALT levels, AST/ALT ratio, GGT levels, TG levels, and FPG levels are given as medians (interquartile range). All *P* values depict significance of differences between Mt5178C and Mt5178A

*BMI* body mass index, *SBP* systolic blood pressure, *DBP* diastolic blood pressure, *AST* aspartate aminotransferase, *ALT* alanine aminotransferase, *GGT* gamma-glutamyl transpeptidase, *TC* total cholesterol, *LDL-C* low-density lipoprotein cholesterol, *HDL-C* high-density lipoprotein cholesterol, *TG* triglyceride, *FPG* fasting plasma glucose, *UA* uric acid

^a^Student’s *t* test

^b^Welch’s *t* test

^c^Mann-Whitney test

^d^Chi-square test

For subjects with Mt5178C, elevated levels of serum AST may depend on coffee consumption (*P* for trend = 0.026) (Table 2). The OR for elevated levels of serum AST was significantly lower in subjects with Mt5178C who consumed ≥3 cups of coffee per day than in those who consumed <1 cup per day (OR = 0.366, 95 % confidence interval (CI) 0.142–0.942, *P* = 0.037). After adjustment for age, BMI, alcohol consumption, habitual smoking, antihypertensive treatment, and antidiabetic treatment, elevated levels of serum AST may also depend on coffee consumption (*P* for trend = 0.013). The OR for elevated levels of serum AST was significantly lower in subjects with Mt5178C who consumed ≥3 cups of coffee per day than in those who consumed <1 cup per day (OR = 0.242, 95 % CI 0.081–0.730, *P* = 0.012). On the other hand, the association between Mt5178A genotype and elevated levels of serum AST does not appear to depend on coffee consumption.

**Table 2:** Odds ratios (ORs) and 95 % confidence intervals (CIs) for high levels of serum aspartate aminotransferase (AST) by Mt5178 C/A genotype and coffee consumption

Genotype and coffee consumption	Frequency (%)		OR (95 % CI)	Adjusted OR^a^ (95 % CI)
	Normal levels of serum AST	High levels of serum AST		
	Serum AST levels <30 U/L	Serum AST levels ≥30 U/L		
Mt5178C				
<1 cup per day	87 (75.6)	28 (25.4)	1 (reference)	1 (reference)
1–2 cups per day	66 (83.5)	13 (16.5)	0.612 (0.295–1.272)	0.490 (0.212–1.134)
≥3 cups per day	51 (89.5)	6 (10.5)	0.366 (0.142–0.942)*	0.242 (0.081–0.730)*
			*P* for trend = 0.026	*P* for trend = 0.013
Mt5178A				
<1 cup per day	49 (79.0)	13 (21.0)	1 (reference)	1 (reference)
1–2 cups per day	56 (82.4)	12 (17.6)	0.808 (0.337–1.934)	0.833 (0.294–2.362)
≥3 cups per day	34 (85.0)	6 (15.0)	0.665 (0.230–1.923)	0.818 (0.231–2.901)
			*P* for trend = 0.438	*P* for trend = 0.656

*P < 0.05

^a^OR adjusted for age, body mass index, habitual alcohol consumption, habitual smoking, green tea consumption, antihypertensive treatment, and antidiabetic treatment

In the case of elevated serum ALT levels, as defined as >30 U/L, for either Mt5178 C/A genotypes, no significant relationship between coffee consumption and elevated levels of serum ALT was observed (Table 3). However, in the case of elevated serum ALT levels, as defined as ≥25 U/L, for subjects with Mt5178C, elevated levels of serum ALT may depend on coffee consumption (*P* for trend = 0.015). The OR for elevated levels of serum ALT was significantly lower in subjects with Mt5178C who consumed ≥3 cups of coffee per day than in those who consumed <1 cup per day (OR = 0.409, 95 % CI 0.202–0.828, *P* = 0.013). After adjustment for age, BMI, alcohol consumption, habitual smoking, antihypertensive treatment, and antidiabetic treatment, elevated levels of serum ALT may also depend on coffee consumption (*P* for trend <0.001). The OR for elevated levels of serum ALT was significantly lower in subjects with Mt5178C who consumed ≥3 cups of coffee per day than in those who consumed <1 cup per day (OR = 0.198, 95 % CI 0.077–1.129, *P* < 0.001). On the other hand, the association between Mt5178A genotype and elevated levels of serum ALT does not appear to depend on coffee consumption.

**Table 3:** Odds ratios (ORs) and 95 % confidence intervals (CIs) for high levels of serum alanine aminotransferase (ALT) by Mt5178 C/A genotype and coffee consumption

Genotype and coffee consumption	Frequency (%)		OR (95 % CI)	Adjusted OR^a^ (95 % CI)
	Normal levels of serum ALT	High levels of serum ALT		
	Serum ALT levels ≤30 U/L	Serum ALT levels >30 U/L		
Mt5178C				
<1 cup per day	82 (71.3)	33 (28.7)	1 (reference)	1 (reference)
1–2 cups per day	56 (70.9)	23 (29.1)	1.021 (0.543–1.919)	0.797 (0.375–1.694)
≥3 cups per day	45 (79.0)	12 (21.0)	0.663 (0.312–1.409)	0.407 (0.148–1.115)
			*P* for trend = 0.346	*P* for trend = 0.090
Mt5178A				
<1 cup per day	41 (66.1)	21 (33.9)	1 (reference)	1 (reference)
1–2 cups per day	51 (75.0)	17 (25.0)	0.651 (0.304–1.392)	0.416 (0.160–1.080)
≥3 cups per day	26 (65.0)	14 (35.0)	1.051 (0.456–2.425)	0.695 (0.249–1.941)
			*P* for trend = 0.953	*P* for trend = 0.285
	Serum ALT levels <25 U/L	Serum ALT levels ≥25 U/L		
Mt5178C				
<1 cup per day	64 (55.6)	51 (44.4)	1 (reference)	1 (reference)
1–2 cups per day	49 (62.0)	30 (38.0)	0.768 (0.428–1.379)	0.556 (0.274–1.129)
≥3 cups per day	43 (75.4)	14 (24.6)	0.409 (0.202–0.828)*	0.198 (0.077–0.512)**
			*P* for trend = 0.015	*P* for trend < 0.001
Mt5178A				
<1 cup per day	32 (51.6)	30 (48.4)	1 (reference)	1 (reference)
1–2 cups per day	38 (55.9)	30 (44.1)	0.842 (0.422–1.680)	0.805 (0.364–1.781)
≥3 cups per day	22 (55.0)	18 (45.0)	0.873 (0.393–1.938)	0.585 (0.212–1.611)
			*P* for trend = 0.701	*P* for trend = 0.353

*P < 0.05; **P < 0.001

^a^OR adjusted for age, body mass index, habitual alcohol consumption, habitual smoking, green tea consumption, antihypertensive treatment, and antidiabetic treatment

In the case of elevated levels of serum GGT, as defined as ≥60 or >51 U/L, for subjects with Mt5178C, elevated levels of serum GGT may depend on coffee consumption (*P* for trend = 0.049 and *P* for trend = 0.015, respectively) (Table 4). The OR for elevated levels of serum GGT, as defined as ≥60 or >51 U/L, was significantly lower in subjects with Mt5178C who consumed ≥3 cups of coffee per day than in those who consumed <1 cup per day (OR = 0.488, 95 % CI 0.240–0.993, *P* = 0.048 and OR = 0.418, 95 % CI 0.209–0.837, *P* = 0.014, respectively). After adjusting for age, BMI, alcohol consumption, habitual smoking, antihypertensive treatment, and antidiabetic treatment, elevated levels of serum GGT, as defined as ≥60 or >51 U/L, may also depend on coffee consumption (*P* for trend = 0.002 and *P* for trend <0.001, respectively). After the aforementioned adjustment, the OR for elevated levels of serum GGT, as defined as ≥60 or >51 U/L, was significantly lower in subjects with Mt5178C who consumed ≥3 cups of coffee per day than in those who consumed <1 cup per day (OR = 0.313, 95 % CI 0.110–0.640, *P* = 0.003 and OR = 0.231, 95 % CI 0.095–0.561, *P* = 0.001, respectively). On the other hand, the association between Mt5178A genotype and elevated levels of serum GGT does not appear to depend on coffee consumption.

**Table 4:** Odds ratios (ORs) and 95 % confidence intervals (CIs) for high levels of serum gamma-glutamyltransferase (GGT) by Mt5178 C/A genotype and coffee consumption

Genotype and coffee consumption	Frequency (%)		OR (95 % CI)	Adjusted OR^a^ (95 % CI)
	Normal levels of serum GGT	High levels of serum GGT		
	Serum GGT levels <60 U/L	Serum GGT levels ≥60 U/L		
Mt5178C				
<1 cup per day	69 (60.0)	46 (40.0)	1 (reference)	1 (reference)
1–2 cups per day	52 (65.8)	27 (34.2)	0.779 (0.429–1.414)	0.575 (0.280–1.177)
≥3 cups per day	43 (75.4)	14 (24.6)	0.488 (0.240–0.993)*	0.313 (0.110–0.640)**
			*P* for trend = 0.049	*P* for trend = 0.002
Mt5178A				
<1 cup per day	41 (66.1)	21 (33.9)	1 (reference)	1 (reference)
1–2 cups per day	36 (52.9)	32 (47.1)	1.735 (0.854–3.528)	1.775 (0.775–4.066)
≥3 cups per day	27 (67.5)	13 (32.5)	0.940 (0.404–2.189)	0.838 (0.320–2.190)
			*P* for trend = 0.911	*P* for trend = 0.711
	Serum GGT levels ≤51 U/L	Serum GGT levels >51 U/L		
Mt5178C				
<1 cup per day	62 (53.9)	53 (46.1)	1 (reference)	1 (reference)
1–2 cups per day	48 (60.8)	31 (39.2)	0.756 (0.422–1.352)	0.569 (0.280–1.155)
≥3 cups per day	42 (73.7)	15 (26.3)	0.418 (0.209–0.837)*	0.231 (0.095–0.561)**
			*P* for trend = 0.015	*P* for trend < 0.001
Mt5178A				
<1 cup per day	37 (59.7)	25 (40.3)	1 (reference)	1 (reference)
1–2 cups per day	32 (47.1)	36 (52.9)	1.665 (0.830–3.339)	1.709 (0.746–3.911)
≥3 cups per day	23 (57.5)	17 (42.5)	1.094 (0.488–2.450)	1.025 (0.412–2.549)
			*P* for trend = 0.673	*P* for trend = 0.988

*P < 0.05, **P < 0.005

^a^OR adjusted for age, body mass index, habitual alcohol consumption, habitual smoking, green tea consumption, antihypertensive treatment, and antidiabetic treatment

## Discussion

In the present study, the joint defensive effect of Mt5178 C/A polymorphism and coffee consumption on the elevation of serum liver enzymes, namely AST, ALT, and GGT, was observed in male Japanese health check-up examinees. Considering that coffee consumption protects hepatocytes from viruses, alcohol, drugs, or other aggressors [1], habitual coffee drinking is expected to exert a desirable effect on the liver in either genotype. For subjects with Mt5178C genotype, more consumption of coffee may reduce the risk of elevated levels of AST, ALT, and GGT. On the other hand, for those with Mt5178A, coffee consumption does not appear to influence abnormally elevated levels of serum liver enzymes.

Several large-scale cross-sectional studies have shown an inverse association between coffee consumption and abnormally elevated levels of serum liver enzymes in populations in Europe [8], the USA [4, 7], and Japan [5, 6]. In the aforementioned epidemiological studies [4–8], age, BMI, alcohol consumption, and habitual smoking were considered to be confounders or effect modifiers. However, genetic factors were not included as confounding factors or effect modifiers in either study. Considering our results, the addition of genetic information may contribute to future epidemiological studies with regard to the effects of coffee consumption on elevated levels of serum liver enzymes.

The genetic frequency of Mt5178C is markedly higher in European, American, and African populations than in Asian populations [28]. This is probably the cause of the inverse association between coffee consumption and elevated levels of serum liver enzyme observed in Europe [8] and the USA [4, 7]. However, we cannot rule out the possibility that coffee consumption alone modulates serum liver enzyme levels, irrespective of Mt5178 C/A polymorphism.

Serum ALT levels are a marker of liver cell injury [29], as well as NAFLD, hepatocellular carcinoma, type 2 diabetes, and cardiovascular disease [30]. In the present study, two different definitions of elevated serum ALT levels, >30 U/L [22] or ≥25 U/L [23], were used. Based on a large-scale community-based study including subjects who consumed <20 g of alcohol per day, Miyake et al. estimated the ATL cutoff levels for NAFLD at 25 U/L for Japanese men [23]. Among Mt5178C genotypic men who are reportedly susceptible to cardiovascular diseases [12–14], the OR for ≥25 U/L of serum ALT, surrogate marker for NAFLD, was significantly lower in subjects who consumed ≥3 cups of coffee per day than in those who consumed <1 cup per day. Moreover, a large-scale population-based prospective study found that elevated levels of serum GGT (>51 U/L in men and >33 U/L in women) are associated with not only liver disease mortality but also mortality from all causes, malignancy, and diabetes [26]. A cross-sectional study showed the association between serum GGT activity and metabolic risk factors for cardiovascular disease among Japanese men [31]. Among Mt5178C genotypic men, the OR for elevated levels of serum GGT was significantly lower in subjects who consumed ≥3 cups of coffee per day than in those who consumed <1 cup per day. Therefore, coffee consumption may have a preventative effect on liver damage, as well as cardiovascular disease, in men with Mt5178C.

Personalized prevention by behavior modification of coffee intake may be more suitable for men with Mt5178C than for those with Mt5178A. Among Mt5178C genotypic men, the risk of hypertension was significantly lower in subjects who consumed ≥2 cups of coffee per day than in those who consumed ≤1 cup of coffee per day [15], and that of abnormal glucose tolerance was also significantly lower in subjects who consumed ≥4 cups of coffee per day than in those who consumed <1 cup of coffee per day [16]. On the other hand, among Mt5178A genotypic men, the risk of hyper-LDL cholesterolemia was significantly higher in subjects who consumed ≥1 cup of coffee per day than in those who consumed <1 cup of coffee per day [17]. Taken together, the present results suggest that coffee intake is more effective at reducing the risk of cardiovascular disease in men with Mt5178C than in those with Mt5178A. However, from the viewpoint of prevention of anemia, coffee consumption may not be recommended for Mt5178C genotypic men [18].

The mechanisms of the joint protective effects of Mt5178 C/A polymorphism and coffee consumption on elevated levels of serum liver enzymes remain unknown. They presumably depend on the biophysical and biochemical differences in the response to certain compounds in coffee between ND2-237Leu and ND2-237Met. NADH dehydrogenase, complex I of respiratory chain, is regarded as the major physiological and pathological site of reactive oxygen species (ROS) generation in mitochondria and itself as a target of assault by ROS [32]. Mouse mitochondrial DNA 4738 C/A (Mt4738 C/A) polymorphism also leads to a leucine to methionine substitution in NADH dehydrogenase subunit 2. ROS production by NADH dehydrogenase is significantly lower in mice with Mt4738A than in those with Mt4738C [33]. Extrapolation from experimental animal models to humans suggests that ND2-237Met suppresses ROS production. Moreover, as methionine residues act as endogenous antioxidants [34], ND2-237Met may also protect NADH dehydrogenase itself from ROS attack. Thus, Mt5178A genotype is thought to be clinically [10–14] and pathologically [33, 34] superior to Mt5178C genotype. Coffee intake exerts antioxidant potential in human subjects [35, 36]. However, the reasons why coffee consumption exerts antioxidant effects on liver in ND2-237Leu genotypic men rather than in longevity-associated ND2-237Met genotypic men remain uncertain. In any case, elucidation of the mechanisms of the combined defensive effects of Mt5178 C/A (ND2-237 Leu/Met) polymorphism and coffee consumption on abnormally elevated levels of serum liver enzymes remains a matter for further biochemical investigation.

Mera et al. reported that the distribution of the AST/ALT ratio by age decade is U-shaped: high in the 1st and 2nd decades, declining to a nadir in the 4th decade, then rising in the 10th and 11th decades [37]. NADH dehydrogenase, centrally regulating the aging process [38], may be relevant to our finding that the AST/ALT ratio was significantly higher in Mt5178C genotypic men than in Mt5178A genotypic men. However, the AST/ALT ratio, clinically useful to assist in differential diagnosis of hepatic disease [39], was not associated with coffee consumption in either Mt5178C or Mt5178A genotypic men (data not shown).

Hepatic status and lipid status may mutually affect one another. NAFLD is clinically associated with detrimental changes in lipid and lipoprotein profiles [40]. On the other hand, statins, lipid-lowering agents, exert substantial improvement in serum liver enzyme levels in patients with NAFLD [41]. Due to unclear data on antidyslipidemic drugs, further research will be required to investigate gene-environment interactions on serum liver enzyme levels after adjustment for statin use. As mentioned above, Mt5178 C/A polymorphism modifies the effects of coffee consumption on serum LDL cholesterol levels [17]. Coffee consumption raises serum LDL cholesterol levels in only Mt5178A genotypic men. This effect may obscure the interaction of Mt5178 C/A polymorphism and coffee consumption on serum liver enzyme levels in men with Mt5178A.

In addition to a lack of information on antidyslipidemic drugs, there are several crucial limitations in this study. First, the study sample was very small. Therefore, it is possible that false-negative results were obtained in statistical analyses for subjects with Mt5178A. We should consider the possibility that elevated levels of serum liver enzymes are modulated by the effects of coffee consumption alone and not by the joint effects of Mt5178 C/A polymorphism and coffee consumption. Second, we analyzed only a single population. Third, this was an exploratory cross-sectional study. To overcome these limitations, a prospective study with a larger sample including a range of ethnic populations is necessary. Fourth, the evaluation of habitual coffee consumption was based on the number of cups consumed per day. Whether there is any interaction between Mt5178 C/A polymorphism and volume of chlorogenic acids, caffeine, or other compounds in coffee on elevated levels of serum liver enzymes warrants further investigation. Moreover, considering that the type of coffee preparation influences serum liver enzymes [8, 42], information regarding the method of coffee preparation will be required. Fifth, because of a lack of data on the amount of alcohol intake, the evaluation of habitual alcohol intake was based on the frequency of alcohol consumption. Alcohol consumption was treated as one of the confounding factors in statistical analysis in this study. The existence of combined effects between Mt5178 C/A polymorphism and volume of alcohol intake on elevated levels of serum liver enzymes warrants further investigation.

## Conclusion

This exploratory cross-sectional study suggests that Mt5178 C/A polymorphism modulates the effects of coffee consumption on elevated levels of serum liver enzymes in male Japanese health check-up examinees. To the best of our knowledge, this is a novel gene-environment interaction on abnormally elevated levels of serum AST, ALT, and GGT levels. Although abstaining from coffee intake is advocated for men with Mt5178C in order to reduce the risk of anemia [18], coffee consumption may be recommended for them in order to reduce the risk of liver injury, NAFLD, hypertension [15], and abnormal glucose tolerance [16]. This genetic information may contribute to individualized prevention of liver injury, NAFLD, and cardiovascular diseases. Moreover, the identification of gene-environment interactions across diverse populations with various lifestyles may contribute to cultural and physiological anthropology [43].

## Abbreviations

ALT, alanine aminotransferase; AST, aspartate aminotransferase; BMI, body mass index; CI, confidence interval; GGT, gamma-glutamyl transpeptidase; LDL, low-density lipoprotein; Mt5178 C/A, mitochondrial DNA 5178 cytosine/adenosine; NAFLD, non-alcoholic fatty liver disease; ND2-237 Leu/Met, NADH dehydrogenase subunit-2 237 leucine/methionine; OR, odds ratio; PCR, polymerase chain reaction; ROS, reactive oxygen species
